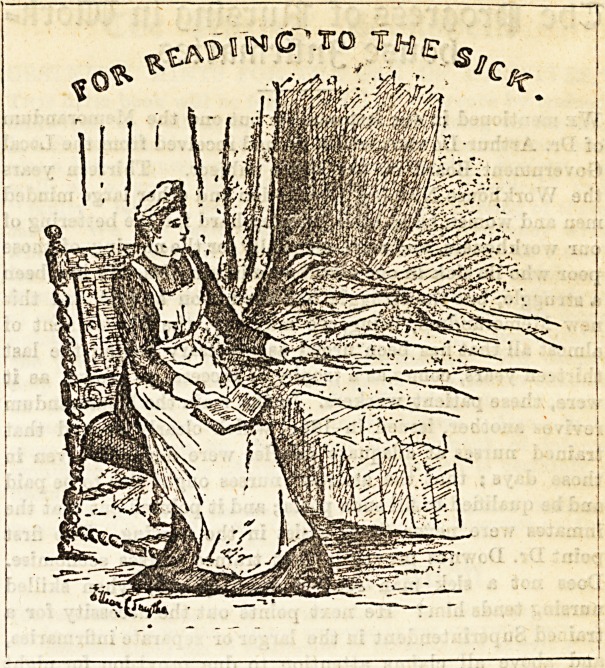# The Hospital Nursing Supplement

**Published:** 1892-06-04

**Authors:** 


### "The Hospital" Nursing Mirror.

Being the Extra Nursing Supplement of "The Hospital" Newspaper.

Contributions for this Supplement should be addressed to the Editor, The Hospital, 140, Strand, London, W.C., and should have the word "Nursing" plainly written in left-hand top corner of the envelope.

### En Passant.

BEHIND GLASS DOORS.—Of all suitable places for the introduction of cupboards with glass doors, surely the hospital ward stands first. We know of one where it is the custom to store the linen within this transparent protection, and the effect is excellent, as well as the absolute insurance of method and neatness. A splint cupboard is a great success when thus treated, and the padding remains in a satisfactory condition when thus efficiently preserved from dust and indiscriminate handling.

WESTMINSTER HOSPITAL SAMARITAN FUND.—We are glad to hear that the new theatre at Queen Anne's Mansions will be opened on June 25th with a Shakespearian dramatic recital, given in aid of this charity. Mrs. Calverley Bewicke's name is a guarantee for an excellent entertainment, and the well-known harpist, Mr. John Thomas, has kindly promised to assist; so, with Archdeacon Farrar in the chair, we may look forward to a successful entertainment.

LAST OFFICES.—It has always been the custom for private as well as hospital nurses to perform the last offices for those patients who die whilst under their care, and it is often a great satisfaction to the friends to know that this sad but necessary work has been done by gentle and reverent hands. Perhaps some of our readers are not aware that it is the custom for many hospitals to allow their private nurses to go, on an emergency, to any household where they may be required (at a moderate fee) for this purpose, and now we find that some nursing institutions adopt the same plan.

AMERICAN NEWS.—Miss Linda Richards has been selected as Directress of the new Methodist Hospital at Philadelphia. Miss Richards graduated in 1873. In 1877 she came over to Europe to study the training school methods, and visited the Nightingale School, the Royal Infirmary, Edinburgh, and afterwards organised the Boston City Hospital School. In 1884 she went to Japan, and organised a training school for Japanese nurses in Kioto, and in March, 1891, she took charge of the Philadelphia Visiting Nurses' Society.— The Illinois Training School has graduated 210 nurses, and there are 118 pupil nurses now in the school. The women of Illinois are going to exhibit a model hospital at the Chicago World's Fair which will be conducted entirely by women. A memorial hospital will shortly be erected in Brooklyn.—"The Trained Nurse" is publishing Dr. Potter's "Ministering Women."

LEICESTER NURSING INSTITUTION.—The twenty-fifth annual meeting of this Institution took place at the Assembly Rooms. Seven more nurses are at work compared with last year. Every year the staff hopes to increase the number of nurses, to meet an ever-increasing demand. This year there is a small fund in hand of the District Nursing account—an unprecedented event. Great efforts are being made to supply some more of the suburbs with district nurses. The Mayor, who presided, said that the Institution was not a profit-getting one, but he thought, inasmuch as it relieved suffering and prolonged life, that it added to the wealth of the borough by these means. The receipts of the private nursing account amounted to £1,420 2s. 10d. We are sorry to see there was an adverse balance, but the illness of several nurses was partly the cause.

NATIONAL HOSPITAL, QUEEN SQUARE. — The Duke of Westminster presided at the annual meeting at the National Hospital, Queen Square, on the 26th ult. The usual business was transacted, and the report read, the accounts being prepared according to the plan which has been proposed for universal adoption by hospitals. The visitors afterwards inspected the new wing, which will shortly be opened for surgical patients, and which contains an admirable clinical theatre and a convenient and well-lighted museum.

PERSONAL INSPECTION.—"No one can adequately comprehend either the need or the value of a hospital without making a *personal inspection*; then he would quickly discern, in the presence of the painful forms of disease which present themselves, in the deplorable accidents which often incapacitate the workman for life, and in the multitude of strange and weary faces which everywhere meet his gaze, that a hospital is not only a necessary factor in civilised life, and an unspeakable boon to the poor, but is also the expression of a Divine and far-reaching sympathy."

THE LADIES' SANITARY ASSOCIATION. — The thirty-fourth annual report tells of much useful work done by the Ladies' Sanitary Association, 22, Berners Street, and it especially calls attention to the encouraging tendency of those who have had the benefit of regular instruction to wish for yearly lectures. There seems some disappointment regarding the sale of the admirable tracts published by the society, and we should strongly advise a special effort this year to attract the attention of the practical section of the public, which is always ready to welcome cheap and sound literature when it is brought prominently before it.

ABERDEEN DISTRICT NURSING ASSOCIATION.—The General Committee has had its first meeting, and has accomplished its affiliation with the Scottish branch of the Q.V.J.I.N. Dr. Angus Fraser has been appointed Chairman, and Miss Katherine Lumsden and Miss Smith, Hospital for Sick Children, have been appointed Honorary Secretaries. Miss Laing, 5, St. Swithin Street, is Honorary Treasurer. Miss Armstrong, of the Q.V.J.I.N., has been secured for the Aberdeen Association, and, being present at the meeting, she received a cordial welcome from the Committee. So far so good, and now a little money would be a most acceptable gift, and would complete the working preliminaries.

RED BLOUSES AND NURSES.—An excellent colour, a cheerful, nay, a national colour, is this fine scarlet hue! Our "red-coats" have won proud mention on many a hardly-fought field of battle, and at the present day some of Her Majesty's nursing Sisters add to their pleasant grey uniform the tiny red cape, which gives such a pretty touch of colour to their serviceable dress. But another kind of garment is in our midst, and we can only hope the fashion of it will be as short-lived as it deserves. We speak of the scarlet blouse just now in favour with a few, and let us hope, a very few, private nurses. Those who wear such startling and slovenly garments must be somewhat destitute of respect for the dignity of their calling, and somewhat deficient in knowledge of the due fitness of things to support that dignity. A blouse is all very well for home wear, for boating, or tennis, but to see a nurse, whose dress should be as consistently modest as her behaviour, arrive to take charge of a patient in such a garment, is really a sight to make angels weep! Such glaring improprieties of costume cannot well be exceeded, although the wearer of a "horsey" collar and manly scarf (which her uniform cloak unfortunately did not hide) is guilty of equally bad taste.

### Ventilation, Disinfection, and Diet.

By P. Caldwell Smith, M.D.

VIII.—DISINFECTION.—*(Continued.)*

Disinfectants — Antiseptics — Bactericides — Deodorants—Disinfection by Heat and Disinfection by Chemicals—Hot-air as Disinfectant, its Disadvantages:—Steam under Pressure—Steam at Ordinary Pressure as Disinfectant—Objections to Use of Steam—Washington Lyons' Apparatus—Goddard, Massey, and Warner's Apparatus—De Meyer's Apparatus—Chemical Disinfectants—Carbolic Acid—Carbolic Oil Useless—Carbolic Powders—Carbolic Soaps.

Although then these are all the germs known to be the cause of disease, we may infer that in small-pox, chicken-pox, scarlet fever, whooping cough, typhus fever, measles, &c., germs are the causal agents, although we are not yet far enough advanced in bacteriological science to demonstrate them.

These germs then being the causes of disease, it is necessary, if we are to prevent them multiplying and causing further extensions of the disease, that something be done to kill them. It is to do this that we use disinfectants.

It is necessary to distinguish between several words, often very loosely used, which will occur in the course of the following remarks: First, a disinfectant is a substance which destroys germ life by its poisonous action. Bactericide means the same thing, while an antiseptic is a substance which arrests the development of germs but does not destroy their vitality. All disinfectants are therefore antiseptics, but all antiseptics are not disinfectants. A deodorant, again, is a substance which kills smell produced by purtifying substances.

Until the experiments of Koch we knew very little as to the real value of the different disinfectants, or as to their mode of action. All the results which he got, as well as those of Drs. Parsons and Klein, are now looked upon as having so far settled the question of the comparative utility of the different disinfectants.

There are two main methods of disinfection. (1) Disinfection by heat, and (2) disinfection by means of chemicals. First as to disinfection by heat. There are three kinds of disinfection by heat; (1) disinfection by hot air; (2) disinfection by steam under pressure; (3) disinfection by steam at ordinary pressure. As to disinfection by hot air, Koch found that germs themselves without spores are destroyed in one and a half hours at a temperature slightly above 212 deg. F. The spores of bacilli required three hours at 284 deg. F. Hot air also penetrates very slowly fabrics such as pillows, even four hours exposure at 284 deg. F. being hardly sufficient to kill the bacilli and their spores. This temperature, moreover, completely destroys the fabrics, so that it would be more efficacious at once to burn them.

The experiments made with steam under pressure (that is to say in a closed vessel) showed that the most resistent spores, viz., those of anthrax, or wool sorters' disease, and those found in ordinary garden earth were destroyed, the former by exposure for ten minutes to a temperature of 212 deg. F., and the latter, which are the most resistent spores we know, by exposure to a temperature of 221 deg. F. for ten minutes.

It was found that exposure to a current of steam at ordinary pressure, that is, at 212 deg., F., the ordinary boiling point of water, was the most satisfactory. Koch says, "It is more certain, more simple, more rapid, more economical, both in original cost and expense of working, and involves less injury to the articles to be disinfected." There are some articles, of course, which should not be exposed to this temperature. Leather articles are at once shrivelled up, and new blankets and flannel lose some of their fineness. Silk and cotton articles are not at all injuriously acted on, nor are dyes very much affected. Exposure to steam at 212 deg. F. may, however, fix stains in materials, that is, if a piece of flannel is stained with blood and put into a disinfecting apparatus, the blood stain is fixed and cannot afterwards be washed out. To prevent this the flannel or other material should be first soaked in cold water.

If an article not of a "fast" colour is put into a steam disinfecting chamber the colour will run, but if this be the case the likelihood is that the article is not of much value and should be burnt.

The most serious objection to this form of disinfection as applied to flannel articles is the loss of elasticity and "fluffiness." The heat removes the natural grease which exists in the wool, and this also explains how blankets will not bear boiling in water.

There are a number of apparatus in the market for the purpose of applying this mode of disinfection. The Washington Lyons' one, used very largely, consists of a large oval boiler made of iron, with double walls and a door at each end. A pressure guage is connected with the casing, and also with the interior chamber, the apparatus being usually worked at a pressure of 10 lbs. per square inch in the casing, and 5 lbs. per square inch in the chamber. When it is to be used steam is turned on from a boiler first into the casing to heat up the chamber. The articles to be disinfected are then placed in the chamber, the doors closed, and steam turned on to fill the chamber, until the first pressure is reached, *i.e.*, 10 lbs. to the square inch. After ten or twenty minutes, according to the size of the articles introduced, the steam is turned off, the doors opened, and the articles allowed to dry.

Another form of apparatus is that made by Goddard, Massey, and Warner. It is of rectangular shape, and a separate boiler is not required, as the lower part of the casing is filled with water, and serves as a boiler. Articles are put in and kept there for ten or twenty minutes, when the steam is allowed to escape and the articles are then dried by a current of hot air, which is drawn through the chamber.

In these two forms of apparatus the steam is employed at high pressure, but in De Meyer's, which is of somewhat similar construction, steam at ordinary pressure is employed.

These forms of apparatus, which are the most efficacious used in this country, are expensive, and require some skilled attention. They ought to be erected at every fever hospital, and all infected articles ought to be sent there by a special conveyance to be disinfected. Of course all the large fever hospitals possess one, but in nearly all the smaller towns in Scotland other and less efficacious means are used, while in some no proper disinfection is attempted.

We now turn to chemical disinfectants, and the first of these we shall mention is carbolic acid. Koch in his experiments found that for complete destruction of the bacteria and their spores seven days was necessary for a 3 per cent. solution, three days for a 4 per cent. solution, and two days for a 5 per cent. For complete disinfection, then, it appears that a 10 per cent. solution of carbolic acid is necessary, or a strength of 1 in 10. This solution would not only be a costly one, but would be destructive to many articles of clothing, as well as to the skin of persons handling it. Even with a 5 per cent. solution, or a solution of 1 in 20, clothes if saturated with it for, say, 36 hours, would be to some extent destroyed. It is also to be remembered that it is frequently impossible to keep an infected article, as, say a chair or bed, in contact with a 5 per cent. solution for this lengthened period. It is necessary then, if carbolic acid be used, that its action should be kept up for 36 hours. This is more necessary if dealing with germs which are difficult to destroy. Thus, for example, it has been shown that the bacilli in expectoration of tubercular patients were not destroyed unless this 5 per cent. solution of carbolic acid be kept in contact with the expectoration for at least 24 hours.

Carbolic acid vapour is comparatively useless at ordinary temperatures, although if used at, say, a temperature of 90 deg. F., it proves efficacious in destroying the germs.

Carbolic oil has been proven to be absolutely useless, and should never be used, as it has no effect at all on the germs beyond that in the oil itself.

There are some carbolic powders in frequent use—Calvert's, which contains selica and alumina; McDougall's, which contains sulphate of calcium, besides the carbolic acid, are not very satisfactory, although Calvert's is the better of the two.

It is useless to use carbolic soaps of any kind as disinfectants, as they contain far too small a proportion of carbolic acid to be of the slightest use.

### Proposed Bill for Registration of Midwives.

THE select Committee met again on May 27th and proceeded with the hearing of the evidence of Dr. Drage, who was positive in his opinion as to country women preferring the assistance of doctors to midwives. He gave the number of births in his own district and other details not affecting the question at issue. He contradicted Dr. Rentoul's figures, which, he said, were not, as asserted, those of Matthews Duncan, whose book he begged the Committee to study for themselves, but we think the gentlemen are better acquainted with medical literature than to search for modern statistics in a book dated 1870. Dr. Athill objected to all registration and declined to allow that any improvement in the status of doctors was due to the medical register. Dr. Hayward gave valuable evidence as to the evil results of untrained women acting as midwives. On May 29th the Committee again sat and received the evidence of Dr. Humphreys, which was very clearly and accurately given, his figures bearing investigation, which was certainly more than could be said of some of the statistics previously offered. Mrs. Malleson (hon. Secretary of the Rural Branch of the Q.V.J.N.), gave an account of the nursing work amongst the poor in the agricultural districts, and spoke highly of their appreciation of trained women, as Mrs. Martin (whom we called Mrs. Malleson by mistake), had also done at a previous Committee meeting. The evidence of Dr. Grailly Hewitt was received with the respect and attention it deserved, for he spoke with the authority of a large experience, and absolutely refuted the assertions of Dr. Rentoul and Dr. Drage that there was not sufficient "material" for the instruction of both students and midwives. His calm and deliberately formed opinions were distinctly in favour of women being thoroughly trained in midwifery, but not in the branches of mediicne and surgery, unless they intended to practise these; he considered the knowledge only acquired by study would be of small value. He also objected to the suggestion of former witnesses that it was proposed to institute a new order of practitioner, as the midwife already existed, and it was desirable to have her as efficient as possible. Dr. Playfair's evidence was the next offered, and amongst other things, he spoke of the present satisfactory work done by certificated midwives, and said he considered the opposition to them was maintained by a very small number of practitioners.

### Wants and Workers.

*Nurse's Cottage.*—A nurse having to go away on account of ill-health, would be glad to hear of any nurses who would like to use her small furnished cottage.—628, Manchester Old Road, Rhodes, near Manchester.

*Nurse Corfmat*, Deptford, sends many thanks to "East Grinstead" and "Stella" for so kindly sending such useful parcels.

### THE RESURRECTION.

SPRING, though tardy, has come at last; the sun shines with power, and everything rejoices under the genial warmth and light of its beams. To many of us it is "the winter of our discontent" which is passing away, for we have pined and wasted with the freezing blasts, that have also bound the earth as with iron; yet now the trees put forth leaves on their gaunt, bare arms, the tender herb is shooting up out of the ground, and Nature speaks loudly of the hope which is hidden in her bosom. The little brown seed has fallen into the ground, to return again, after many days, as "bread to strengthen men's hearts." The chrysalis lies buried for months, but rises with the glorious wings of the butterfly, true type and beautiful emblem of the soul which can never die.

This lesson of the Resurrection of the dead comes with power and conviction to our hearts as we look around us, for many of our loved ones have passed away, gone over to the majority; shall we then sorrow for them as those that have no hope? No. "If we believe that Jesus died and rose again, even so also them that sleep with Jesus will God bring with Him." The wisest of the heathen took lessons of comfort from the yearly revival of tree and shrub and insect life, but it is the Christian alone who can face death bravely, for his trust is in the Mighty Conqueror who has put all things in subjection under His feet.

Some of us may die in the near future, all of us must one day pay the debt of nature, but whether sooner or later, let us pray that it may be only a going to our rest, a falling asleep in Jesus, for then death will have no sting. Christ is the Resurrection and the life, whosoever believeth in Him, though he were dead, yet shall he live, and whosoever liveth and believeth in Him shall never die. Shall we not rise again a glorious and spiritual body?

"On the Resurrection morning
Soul and body meet again;
No more sorrow, no more weeping,
No more pain!"

Soul and body have been parted for awhile, the flesh has kept its Sabbath waiting in a holy stillness, "with feet toward the morn." The soul has been contemplating with earnest prayer the perfections of God, but now reunited, they wake up after Christ's likeness, and are "satisfied with it."

To that brightest of all meetings
Bring us, Jesu Christ, at last.

### The Progress of Nursing in Workhouse Infirmaries.

We mentioned in our last number but one the Memorandum of Dr. Arthur Downes which we had received from the Local Government Board on the above subject. Thirteen years the Workhouse Nursing Association and other large-minded men and women have been working hard for the bettering of our workhouses, and more especially for the nursing of those poor who lie sick in our workhouse infirmaries. It has been a struggle, but the struggle has been won at last, and this new Memorandum, being, we may say, the embodiment of almost all that has been urged as necessary during the last thirteen years, comes as a trophy of success, to crown, as it were, these patient workers. In the main the Memorandum revives another, issued in 1865, which clearly showed that trained nurses at adequate salaries were desirable even in those days; that the assistant nurses ought also to be paid and be qualified to fill such posts; and it pointed out that the inmates were never fit to assist in the nursing. The first point Dr. Downes proves is that trained nurses economise. Does not a sick man recover more quickly when skilled nursing tends him? He next points out the necessity for a trained Superintendent in the larger or separate infirmaries, and above all claims attention to due provision for night nursing.

The 1865 Circular pointed out the desirability of a separate nursing home, removed from the distraction of a large workhouse. "Nursing," says Dr. Downes, "must, like every business, be learned, and by establishing a well-considered system of nursing, the guardians of a large workhouse may, in due course, train their own nurses and assist in supplying a demand which is certain to increase."

These are the principal points of the Memorandum, and then apart from the diminution of suffering which trained nurses would cause there is the financial view of the matter to be considered as follows: Salaries, uniforms, rations, and quarters, against efficiency, saving in waste, saving in wear of appliances, detection of malingering, curable cases more quickly fit for discharge, increased sick ward accommodation, training of probationers. And now it remains for guardians to remember their responsibilities; we have this year such an increase of lady guardians that if they work as they should and can in informing themselves of the possibilities the law allows them, improvements ought to come quickly. The former Memorandum became in many places so much waste paper, its wise suggestions were left unnoticed, because guardians could not or would not do their duty, but happily Dr. Downes' report is issued in better days, when public opinion is better educated, and when dark corners and bad administration have to submit to a keen, searching criticism.

The meeting of workhouse infirmary nurses last week showed what bright, capable women have come forward ready to take up this branch of nursing; they are as interested and as satisfied about their work and as happy with "our mother"—as many of them called their Matron—as any nurse at a regular hospital.

In a short address which Dr. Savill, Medical Superintendent at Paddington, gave to the nurses, he spoke of the desirability of every large workhouse infirmary possessing a training school of its own, and the excellence and thoroughness of the training in many of the infirmaries, the regularity of the work and the experience of managing patients, and the thorough grounding in nursing generally which nurses obtain in these institutions; he also gave two pieces of advice which were meant more especially for infirmary nurses, but which would not come amiss to any of us. "The first is to cultivate a personal relationship and sympathy with all around you, with your patients, with your doctor, and with your manageress. Have you not noticed the difference in 'tone' there is in the wards? In the well-ordered ward the Sister is in sympathy with each and all, she enters into trials and joys, and in that way she possesses a moral influence, and she is obeyed because she is loved. You must all try and remember that you are a part of the institution you work for, and the other suggestion that I wish to make is this, to set a high ideal before you, but do not be disheartened if you do not achieve it all in one day. Cling hard to your ideal, and each day chronicle some little abuse corrected, some little improvement made." And this is all true: guardians may do much, and nurses by their heart and soul work may be conscious of participating in a fight worth winning. Will not our richer friends and acquaintances come forward and send some additional monetary help to the Workhouse Infirmary Nursing Association, 6, Adam Street, Adelphi, Strand?

### A Letter from Fiji.

Suva, Fiji, March 31st, 1892.

Ever since I have been out here I have purposed writing to you, but the climate and circumstances in general have been somewhat unfavourable to correspondence, leading me to confine mine to such as was indispensable. As, however, the appointment of Matron-Nurse will be vacant ere long, some little account of the place and a few hints may be interesting to those who think of applying for it.

The hospital is situated at the top of a steep hill, about a mile and a-half out of the town of Suva and beyond it lie the gaol, the Immigration Dépôt, the cemetery, and at a short distance in another direction is the Lunatic Asylum. The Hospital is kept up entirely by the Government and is under the Chief Medical Officer of the colony. The District Medical Officer for Suva and Navna resides on the premises and is styled the Resident Medical Superintendent; he has absolute control over every person and thing in the institution subject, of course, to the approval of the Chief Medical Officer. The Matron-Nurse has charge under him of the housekeeping and nursing. There are a Fijian head warder and assistant warder. The head warder's wife acts as female warder and as laundress. There are seven native students who live on the premises; they dress the cases and make up medicine, &c., under the supervision of the European dispensing chemist, who is also resident. The ward work, cooking, gardening, fetching provisions, &c., from town, and part of the washing is done by Indian, Fijian, and Polynesian convicts of good conduct and long sentence; of them there are nine. This last arrangement sounds rather curious at first, but in practice it works well, and they are far more to be depended on than paid native labour; the Indian prisoners in particular often make very good nurses. The patients are of various nationalities—European, Half-caste, Fijian, Rotuman, Samoan, Tongan, Polynesians from all parts, Indian coolies, and Madrasis. They vary in number from about forty to seventy, the average being sixty; but we seldom have more than one or two Europeans at a time, and are often without any at all. The native cases, Fijian especially, are usually slight, being mainly cases of strumous ulceration, lupus, or elephantiasis. Polynesian and Indian cases are generally medical and of a more serious character. Dysentery, pernicious anæmia, and chest complaints are very common and very fatal among them. Few of the patients or warders speak English, so that sufficient Fijian for conversational purposes is requisite, and many people find little difficulty in picking it up.

The hospital buildings are all a little way apart and not connected by any covered way, but by rough paths paved with blocks of coal, which are rather awkward on dark and wet nights and most destructive to shoe leather. Kid boots and shoes do not stand the climate well; neither does woollen clothing. Ants, cockroaches, and moths abound, and have a penchant for wools and shoe leather. I brought out caps with me, but have never worn them, as I found a hat, plus an umbrella, necessary whenever I stepped outside my own little house. The work is not heavy, but sufficiently trying on account of the constant heat, and natives as servants are also rather trying to European patience, for they are so slow as a rule.

There is an exquisite view across the bay from the hospital, and all the surroundings are very pretty. The Matron's quarters are comfortable, and the medical officers are most kind and considerate and pleasant to work with, but, of course, the great drawback to the appointment is its isolation, and the loneliness at times is apt to be depressing.

People out here dress very well, and much as they do in a hot summer at home. In spite of the cockroaches' depredations woollen stockings are better to have than cotton ones, as they are a greater protection from mosquitoes, and are, I find, more comfortable to wear. A lady who rides at all will probably find a habit useful, and also a thin waterproof; but she must not do as I did, and provide herself with a bathing dress! She will never have an opportunity of using it. Silk gloves are more useful than kid, except for riding; but there are shops out here where all such things can be bought, only it is a very expensive place, everything being at least double the price it is in England. A sewing machine and a dress would be more useful as the dressmakers are expensive and not over satisfactory.

### Appointments.

Central London Sick Asylum.—Miss Elina M. Smith has been appointed Matron at this asylum, and entered on her duties on May 19th. Miss Smith trained at St. Bartholomew's Hospital, and afterwards went on the permanent staff of that institution. She was next appointed Sister at the York County Hospital, and became "Home Sister" at Great Ormond Street in 1881. In 1890 she became Assistant Matron at Paddington Infirmary, and was afterwards Matron at the Samaritan Hospital for Women at Nottingham, which post she held until the present time.

Royal South Hants Infirmary, Southampton.—Miss Mollett has been appointed Matron of the Royal South Hants Infirmary, Southampton. Miss Mollett was trained at St. Bartholomew's Hospital, and was afterwards Sister at the Children's Hospital, Shadwell. She was next Superintendent of nursing at the National Hospital, Queen Square, and Matron to the Chelsea Infirmary.

Ramsay Hospital, Naini Tal.—Miss Georgina Macvitee has been appointed Lady Superintendent of the new Ramsay Hospital at Naini Tal. Miss Macvitee was trained at St. Bartholomew's Hospital and afterwards went out to India and joined Lady Roberts' Nursing Staff, and for the last five years has been doing good and real hard work in the military hospitals pioneering for the Government Sisters at Meerut, Bareilly, and other important stations, and winning for herself by her zeal, kindness, and goodness of heart the gratitude and esteem of all with whom she came in contact. Miss Macvitee has just commenced her new duties at Naini Tali, and we wish her every success in her new undertaking.

### Hints to Nurses.

Petrola.—A great benefit is conferred on those who have to perform the unsavoury task of washing surgical bandages by the inventor of "Petrola," the "washer, cleanser, and disinfector." We can state, from personal experience, that it not only removes stains and grease from linen and other materials, but that it greatly facilitates the labours of the laundress. Anyone practically interested in washing or cleaning work, should send for a sample of "Petrola" from the agent, W. Mann, 129, Livingstone-road, West Brighton.

### The Nurses' Bookshelf.

### OBSTETRIC HINTS FOR THE USE OF MIDWIVES.*

This little book will be found useful for reference by trained midwives, as well as by nurses who are going in for the L.O.S. examination. It contains various illustrations, and also a list of questions given by the Obstetric Society, as well as a great deal of information on "natural" and "faulty" labours, and the attention to and nursing of these cases. We are particularly glad to see the frequency with which the writer gives the advice to the midwife to "send for assistance" in all those difficult situations which are fraught with danger to mother or child. We are ourselves of opinion that the better trained a woman is, the more quickly does she foresee the emergency which necessitates the presence of a duly-qualified practitioner. We cannot help wishing that space had been given for a longer chapter on the all-important subject of antiseptics, of which the value can never be too strongly insisted upon. Also a little more information on the proper nourishment of infants would be a welcome addition to a book which is certainly a useful one.

* "Obstetric Hints for the Use of Midwives." By R. J. Maitland Coffin, F.R.C.P.Ed. Price 2s. (Publishers, Baithby, Lawrence, and Co.)

### Everybody's Opinion.

*[Correspondence on all subjects is invited, but we cannot in any way be responsible for the opinions expressed by our correspondents. No communications can be entertained if the name and address of the correspondent is not given, or unless one side of the paper only be written on.]*

### OFF DUTY.

"E. F. G." writes: Those nurses to whom the "Nursing Mirror" has sent tickets of admission for the Botanical and Zoological Gardens, express themselves as greatly pleased by the kind thought. They venture to hope that "Fellows" of these societies will, from time to time, place it in the editor's power to give them the enjoyment which is always associated with a stroll in these beautiful grounds.

### PRIVATE NURSES AND THEIR WAYS.

"V. M. H." writes: How is it we are always hearing complaints of private nurses nowadays? Is it that women who behave themselves in hospital find a little sole responsibility turns their heads? I have heard some very hard things said lately about private nurses. "They may be all very well in a big house with plenty of servants, but in a small house they are more bother than enough." Another lady had two nurses to attend her, but as the nurses were continually retiring to gossip in the adjoining room, she could have dispensed with their services. I have no doubt that there is a good deal of exaggeration, but it is a disgrace that there should be even a whisper of bad conduct. In my own experience I have found nurses sometimes too imperious; they dash up the window when the room requires airing, and dogmatise about fresh air when gentle persuasion would work much better. What sounds nothing to a healthy person seems cruelty to a sick person, and people ought to send honest reports on their nurse's conduct to the Lady Superintendent, and if, when the complaint is found just and reasonable, the Superintendent will do nothing to prevent its recurrence, it is about time to show up that institution.

### Notes and Queries.

### Answers.

*Olaf.*—You must send name and address in accordance with our rules, when we shall be glad to send information.

*An Anxious Learner.*—You can learn massage at the Birmingham Home of Maasage. 3, Great Charles Street, Birmingham.

*Sister F.*—A district nurse's outfit should contain: 1 thin and 1 warm cloak or ulster, 1 bonnet, 4 print dresses, 24 aprons, and 2 pairs of well-made boots or shoes. She will also need a good stock of collars and cuffs, and a leather bag to take "dressings" and other necessaries on her rounds. She should avoid too heavy a bag, whilst selecting one sufficiently large to contain her stock of useful things. In bad weather a stalking cap, tied down over her ears, and an ulster is by far the most sensible attire. If the distances necessitate a pony and cart, a waterproof driving coat, large enough to go over a coat, and a waterproof rug are absolutely necessary.

### Off Duty

### Tired of Life.

*(Concluded from page lx.)*

Throughout the close of that day Mrs. Daley seemed to improve, and during the night she enjoyed several hours of undisturbed sleep. It was not until

"The morn came, dim and sad
And chill with early showers,"

that the fatal change appeared. Waking from her sleep, she complained of feeling faint and cold. I at once employed the usual restoratives, but finding no improvement in her state, roused her husband, and begged him to go for Dr. Wilmot, who, when he had examined his patient, said—

"You did right to send for me. She is very bad indeed; but you can do no more than continue the remedies you have already used. I will come again in an hour."

Mrs. Daley now became rapidly worse. Her strength visibly declined, and she soon became semi-delirious. Leaning over her I several times caught these, and similar words: "Charlie's pocket-book—hide it away—no one must know! O, Charlie! Ruin—ruin!" And again, uttered in a faint, but heartrending tone, "Poor baby—father—disgrace!"

O course, I attached but little importance to these words at the time, but later on they acquired a deeper significance.

As the hours passed slowly on, it was evident that life was ebbing fast. The poor husband hung, heart-broken, over the wife, who would never recognise his face or voice again. At the last moment consciousness returned, and as the vital spark flickered, expiring in the socket, her fading eye met mine, and she whispered, "Nurse, I am going now—where the wicked cease from troubling, and the weary are at rest."

These were her last words. Then

"Her quiet eyelids closed; she had
Another morn than ours."

"Failure of the heart's action," Dr. Wilmot said, as he looked upon the quiet form, almost as much shocked as we were at this sudden and unexpected death. Yes, but what could have been the secret sorrow which had pressed so sorely on that heart?

We laid her to rest—not merely the rest of the worn-out body, but the repose of the soul for which she had longed—on the following day, amidst the almost uncontrollable grief of her desolate husband, and the sincere regret of those few friends and acquaintances who had learned to know and esteem her.

Their pretty child was sent by its father as soon as possible to the care of his wife's relatives in England, and for some time I heard no more of the family. Life passed on, bringing, as it does to all, but especially the doctor and nurse, new duties, with their pressing demands on time and energy, to lessen, if not obliterate, former interests and sympathies. I might never have known more of my patient, nor have penetrated into the secret which had poisoned her life, had it not been for a startling disclosure, which, for a brief space, convulsed the little world of Maritzburg.

About six months after his wife's death it began to be whispered that Mr. Daley was getting into difficulties. Shortly after this it became known that, pleading a fit of indisposition which made him unfit for the task, he had requested a friend to go through his books for him. What prompted the unfortunate man thus to court certain detection was never known, but the inevitable result followed. His friend, who was himself largely concerned in some of the schemes with which Mr. Daley was connected, had not proceeded far in his work before he found ground for suspicion; suspicion soon became certainty, and it was his painful duty to make known the fact that Mr. Daley had been carrying on an extensive course of fraud and embezzlement. Scarcely had the community recovered from the shock of this discovery, when a darker tragedy occurred. The miserable man had found means to commit suicide in gaol while awaiting his trial, and thus died, after having caused distress and even ruin to many innocent persons, for it appeared that forged scrip to an almost inconceivable extent had been issued by him. The only wonder was that he had escaped detection so long. He left a letter for his friend, in which he said that, from some words spoken by his wife on her death-bed, he knew she had discovered his guilty secret; and this knowledge, with the remorse it occasioned him, led to his present course. He had determined to throw everything up and put an end to the life which had become a hateful burden. During an investigation at his house a pocket-book was found in a secret drawer, in which he had been in the habit of making—with the clumsiness which sometimes characterises even clever criminals—entries that must have led to his detection had it fallen into the hands of the most unsuspicious person. Everything was now clear to me. I understood the hopeless depression of my unhappy patient, and her longing to quit a world in which her dearest hopes and affections had been so cruelly blighted. I recalled her dying words, and knew now that there was method in their madness. "Charlie's pocket-book—no one must know! Oh, Charlie! Ruin, ruin!"

It was evident that his pocket-book—no doubt, through some trivial accident—had revealed the fatal truth to her; that with her timid and reserved disposition she had lacked the courage to tell her husband he was now seen in his true character, while the terrible secret had preyed upon her mind and rendered her an easy victim to disease, which she had neither strength nor wish to resist.

A braver woman might, perhaps, have faced the worst. She might have sought out some means of escape and of redemption for the father of her child, and the husband whom she must still love and pity if she could no longer esteem. But Alice Daley was of different calibre. Too early crushed by sorrow and shame, she had longed only for release, and for that rest which alone could heal her deceived and broken heart.

Some time after the occurrence of these painful events, having a few days at liberty, I one evening took a quiet walk to the cemetery, where several of my patients and friends lie buried. It is a beautiful spot, lying just outside the city. Beyond it is the park, with its background of open country, green hills, and swelling valleys, over which a cool "coast breeze" is always blowing. The burial ground itself is very tastefully laid out, and is bright with many trees and flowers unknown in England.

I soon found Mrs. Daley's grave, which her husband's care had already made a beautiful spot. He was never to lie beside her in the last sleep, but loving and remorseless hands had done what they could for *her* resting place. Yes, with all his faults he had loved her fondly; perhaps that very love had caused the first temptation to wrong-doing, through the mistaken desire to surround her with every luxury and refinement. Had she been cast in a stronger mould, might she not have reclaimed him? But the battle of life had been too hard for her, and as I lingered beside her grave and repeated her last words, which her husband had recorded on the simple stone, I felt thankful that for over-weighted hearts like hers, as well as for those who have bravely borne the burden and heat of the day, there is a merciful rest provided "where the wicked cease from troubling."

## Figures and Tables

**Figure f1:**